# A review of prostate cancer treatment impact on the CNS and cognitive function

**DOI:** 10.1038/s41391-019-0195-5

**Published:** 2019-12-16

**Authors:** Charles Ryan, Jeffrey S. Wefel, Alicia K. Morgans

**Affiliations:** 1 Division of Hematology, Oncology and Transplantation, University of Minnesota, Minneapolis, MN USA; 2 The University of Texas MD Anderson Cancer Center, Houston, TX USA; 3 Feinberg School of Medicine, Northwestern University, Chicago, IL USA

**Keywords:** Prostate cancer, Cancer

## Abstract

**Background:**

Androgen deprivation therapy (ADT) is the backbone of systemic therapy for men with prostate cancer (PC); almost one-half of patients receive treatment during their disease course. However, a range of cognitive and other central nervous system (CNS) changes have been associated with ADT. In this review, we discuss extant data describing these complications and the mechanisms through which medications used to deliver ADT may affect them.

**Methods:**

We performed a MEDLINE search for appropriate papers published between January 2000 and December 2018. Relevant papers were selected and reviewed; additional publications were identified by manually assessing references from included papers, and recent congress abstracts.

**Results:**

Of ~230 search outputs, 33 were selected for inclusion. Some studies suggested a clear association between ADT and CNS effects in men with PC, whereas others did not. Accurate assessment is limited by test instrument variability, inadequate sample sizes, short follow-up duration, and limited prospective longitudinal studies. The approved second-generation androgen receptor (AR) inhibitors enzalutamide and apalutamide were associated with some CNS-related adverse events (AEs) in clinical studies, including fatigue (which can interfere with cognitive function). The androgen synthesis inhibitor abiraterone acetate was associated with a low CNS AE profile when compared with enzalutamide. The AR antagonist darolutamide demonstrated a comparable incidence of cognitive disorder in clinical trials to that of ADT alone.

**Conclusions:**

Adequately caring for men receiving ADT requires an understanding of the symptoms, incidence and magnitude of cognitive effects, and a feasible approach to cognitive assessment and management in clinical settings. Some CNS effects could relate to blood–brain barrier penetration and direct AR inhibitor activity; drug safety profiles may differ by the degree of blood–brain barrier penetration of particular agents. Ongoing clinical trials seek to define the CNS tolerability of newer AR pathway-targeted therapy options more clearly.

## Introduction

Prostate cancer (PC) is the fifth leading cause of cancer-related death in men worldwide, with an estimated 307,000 deaths in 2012 (6.6% of total male deaths) [1, 2]. Androgen deprivation therapy (ADT) is the backbone of systemic therapy for men with PC, with 44.8% of patients receiving ADT during the first year after diagnosis in one US study. This increased to 48.7% in men aged ≥75 years who had T1/T2 tumors with high-grade histology, or T3/T4 tumors [3–7]. With prolonged exposure to ADT, many patients develop castration-resistant PC (CRPC) driven by one or more resistance mechanisms, typically involving retained and enhanced androgen receptor (AR) signaling [8]. The recognition that CRPC continues to be driven by the AR axis led to the development of novel AR pathway-directed therapies, such as abiraterone acetate (approved for metastatic CRPC [mCRPC] in the US, European Union, and Japan) [9–11], enzalutamide (approved for mCRPC in the US, European Union, and Japan; also approved in the US for nonmetastatic [nm]CRPC) [12–14], apalutamide (approved in the US, European Union and Japan for nmCRPC) [15–17], and darolutamide (approved in the US for nmCRPC) [18]. Although generally well tolerated, evidence suggests that ADT may exert negative effects on cognitive function and affect the central nervous system (CNS) [19, 20]. This is consistent with data associating low serum testosterone with dementia risk in aging men without PC [21]. Second-generation AR inhibitors, such as enzalutamide and apalutamide, may also affect CNS function, as seizures have been observed in clinical trials of both therapies [22–24]. Clinical trials of enzalutamide, abiraterone acetate, and apalutamide in patients with CRPC have reported CNS-related adverse events [22–28]. Falls have also been reported as a CNS-related event in patients with metastatic PC receiving treatment with abiraterone acetate (5.9%) and enzalutamide (4.6%) in pivotal, phase 3 registration trials, and in a large retrospective observational study [9, 12, 23]. In the PROSPER (enzalutamide) and SPARTAN (apalutamide) trials in patients with nmCRPC, the incidence of falls in the active treatment groups was 11% and 15.6%, respectively [24, 28]. However, the etiology of falls in patients receiving AR inhibitor therapy has yet to be fully elucidated. CNS-related adverse events may lead to an increased morbidity, reduced quality of life (QoL), decreased efficacy of cancer treatment due to dose interruptions or reductions, or diminished treatment adherence [23].

Given these factors, awareness and identification of neurological complications by practitioners is critical as a first step toward mitigating these adverse events.

The objective of this review is to evaluate the effects of currently available AR-targeted therapies on the CNS and cognitive function and discuss the mechanisms by which they may occur.

In the context of this review, we define “cognitive function” as mental processes, such as memory, learning, reasoning, and attention, as opposed to other CNS effects, i.e., fatigue, seizure, falls, anxiety/depression, insomnia, headache, restless leg syndrome, presyncope, insomnia, dizziness, asthenia, which may occur independently of effects on cognitive function.

## Literature search

We performed a manual MEDLINE search using the following terms separately or in combination: prostate cancer, androgen deprivation therapy, androgen receptor, central nervous system, and cognitive function. Only English language articles were included, and the search was limited to articles published between 01/2000 and 12/2018. Relevant papers were selected and reviewed based on their abstracts; after duplications, a total of 33 papers were identified for inclusion. Additional supporting literature was identified by manual searches of references of included papers (nine in total) and recent congress abstracts (six in total).

## ADT-mediated CNS effects

The extent to which ADT is associated with cognitive changes and other CNS effects in men with PC is unknown and controversial [19, 20, 29–32], with some studies suggesting a clear association and others finding none. One potential reason is the confounding of non-ADT-associated aging-related hormonal changes. In non-PC populations of aging men, low levels of free testosterone have been associated with reductions in visual and verbal memory, processing speed, and visuomotor and spatial ability, particularly in men aged >70 years [33–37]. Anatomical studies demonstrate wide distribution of AR expression in the brain, with the greatest expression present in the hippocampus and amygdala, areas associated with memory, emotional processing, and libido, among others. The neurological changes associated with androgen deprivation occur in the same regions affected by age-related decline and are consistent with our knowledge of loci of AR expression [38, 39]. Unsurprisingly, significant cognitive declines in visuospatial ability, visuomotor tasks, and executive function have been reported in patients with ADT-treated PC [19, 20, 40].

McGinty et al. performed a systematic review of data from 14 studies that investigated cognitive function in patients with nonmetastatic or metastatic PC receiving ADT compared with healthy men or men with PC not receiving ADT. The findings showed that ADT significantly reduced visuomotor ability (effect size –0.67, 95% confidence interval [CI] –1.17 to –0.17; *P* = 0.008), but not other cognitive domains [19]. The duration of ADT treatment at the time of follow-up was a significant moderator of the effect of ADT on visuomotor ability, with a larger magnitude of deficits seen in studies with a shorter time to follow-up [19].

Several studies have failed to reveal associations between ADT and cognitive change (Table 1). A prospective controlled trial (PCT) by Alibhai et al. used a battery of 14 neuropsychological tests in eight cognitive domains but found no consistent evidence of adverse effects on cognitive function based on 12 months of ADT use in elderly men with PC. In adjusted regressions, ADT use was associated with worse immediate memory (*P* *=* 0.029), working memory (*P* *=* 0.031), and visuospatial ability (*P* *=* 0.034), but other analytical approaches did not confirm these findings [30]. In a cross-sectional study of 57 patients with nonmetastatic PC and 51 age-matched controls, ADT was associated with fatigue, low energy, poor bladder control, and sexual dysfunction, but no between-group difference was observed in cognitive function [29]. Another PCT compared patients with nonmetastatic PC starting continuous ADT, patients with nmCRPC not receiving ADT, and healthy controls. Twelve months of ADT were not found to be associated with changes in self-reported cognitive concerns, using the Functional Assessment of Cancer Therapy-Cognitive Function (FACT-Cog) assessment tool [41]. However, data obtained from patient-reported outcome (PRO) measures should be considered with care. PROs have not been validated as a means to assess cognition. They are subjective, based on personal perceptions of cognitive function, and may be affected by factors such as mood and fatigue. Objective tests remain the gold standard for measuring cognitive function—allowing the identification of treatment-related cognitive issues that may impact daily life. However, PROs do provide a useful measure of patient perceptions of impairment and its impact on QoL [42, 43]. One population-based analysis included 101,089 men (15,748 with PC receiving ADT, 34,865 with PC not treated with ADT, and 50,476 without cancer) and used Medicare claims linked with Surveillance, Epidemiology, and End Results (SEER) data to assess whether ADT exposure was associated with one of the several cognitive diagnoses in men with ADT-treated PC compared with men with PC not treated with ADT [44]. The authors reported that ADT was not associated with an increased risk of cognitive disorders compared with patients with PC who had not received ADT in the population overall (risk ratio 0.99; 95% CI 0.94–1.04) [44]. A systematic review and meta-analysis of cognitive impairment in men receiving ADT for PC also found no statistically significant risk of overall cognitive impairment after ADT [45].

**Table 1:** Studies of ADT-mediated cognitive effects.

Study details	Study type	Domains assessed and measurement method
Controlled comparative study (58 patients on ADT; 84 prostatectomy only; 88 healthy controls) [20]	Prospective controlled trial	• Verbal memory: HVLT-R, WMS-III Logical Memory I & II • Visual memory: BVMT-R • Attention: Color Trails 1, WMS-III Digit Span, WMS-III Spatial Span, SDMT • Executive function: Color Trails 2, COWA, TIADL
Cross-sectional study (57 patients on ADT; 51 healthy age-matched controls) [29]	Prospective controlled trial	• Physical tests: 6MWT • HRQoL, fatigue, self-reported cognitive function: FACT general version 4 (fatigue and cognitive modules); FACT-Cog • Neuropsychological: Folstein Mini Mental Status; High Sensitivity Cognitive Screen
Controlled comparative study (77 patients on ADT; 82 not on ADT; 82 healthy controls) [30]	Prospective controlled trial	• Attention (Digit and Spatial Span Forward), processing speed (TMT A), Verbal Fluency (COWA, Animal Fluency), visuospatial ability (Card Rotations, JLO), verbal learning and memory (CVLT), visual learning and memory (BVMT-R), executive functions, working memory (Digit and Spatial Span Backward, SWMT errors, CALT), executive functions, cognitive flexibility (TMT B, D-KEFS, Color Word Interference Test)
Controlled comparative study (81 patients on ADT; 84 not on ADT; 85 healthy controls) [41]	Prospective controlled trial	• Self-reported cognitive function: FACT-Cog scales PCI and IPCIQOL
Retrospective cohort analysis of medical record data (2397 patients on ADT) [32]	Population-based study	• Alzheimer’s disease onset: based on terms from clinical notes and ICD-9 diagnostic code 331.0
Retrospective cohort analysis of SEER-Medicare population data (50,613 with PC; 50,476 without) [44]	Population-based study	• Proportion of patients with a depressive, cognitive or constitutional disorder following PC diagnosis/study entry. Comparison by *X*^2^ test
Retrospective cohort analysis of medical record data (9272 patients with PC, 1826 of whom received ADT) [46]	Population-based study	• New-onset dementia: based on terms from clinical notes and ICD-9 diagnostic codes 290.0–290.9, 331.0–331.2, or 294.1–294.21
Retrospective cohort analysis of medical record data (35,401 patients with PC, 24,567 of whom received ADT) [47]	Population-based study	• Cognitive dysfunction—defined as “the loss of intellectual functions, such as thinking, remembering, and reasoning, with sufficient severity to interfere with daily functioning, including dementia or AD”. Identification was using ICD-10 diagnostic codes
Systematic literature review (14 studies, 417 ADT-treated patients) [19]	Systematic review and meta-analysis	• Studies must have reported objective neuropsychological data • Included tests were divided into seven cognitive domains: attention/working memory, executive functioning, language, verbal memory, visual memory, visuomotor ability, and visuospatial ability
Systematic literature review (two prospective, four retrospective cohort studies, 442 and 67,644 patients, respectively) [45]	Systematic review and meta-analysis	• Prospective studies based on ICCTF criteria (impaired cognitive performance defined as ≥1.5SD below published norms on ≥2 tests, or ≥2.0SD below published norms on ≥1 test) • Cognitive domains assessed (prospective trials): attention, processing speed, verbal fluency, learning and memory, visuospatial ability, executive function, cognitive reserve

*6MWT* 6-Minute Walking Test, *ADT* androgen deprivation therapy, *BVMT-R* Brief Visuospatial Memory Test–Revised, *CALT* Conditional Associative Learning Test, *COWA* Controlled Oral Word Association, *CVLT* California Verbal Learning Test, *D-KEFS* Delis-kaplan Executive Function System, *FACT-Cog* Functional Assessment of Cancer Therapy-Cognitive Subscale, *HRQoL* health-related quality of life, *HVLT-R* Hopkins Verbal Learning Test–Revised, *ICCTF* International Cognition and Cancer Task Force, *IPCIQOL* Impact of PCI on Quality of Life, *JLO* Judgment of Line Orientation, *PC* prostate cancer, *PCI* perceived cognitive impairment, *SD* standard deviation, *SDMT* Symbol Digit Modalities Test, *SEER* Surveillance, Epidemiology, and End Results, *SWMT* Spatial Working Memory Task, *TIADL* Timed Instrumental Activities of Daily Living, *TMT* Trail Making Test, *WMS* Wechsler Memory Scale

Other studies do report such associations (Table 1). A prospective clinical trial included 58 men with PC initiating ADT, 84 men with PC not receiving ADT, and 88 age- and education-matched controls without PC [20]. At 12 months of follow-up, a significantly greater proportion of men treated with ADT were categorized as having cognitive impairment when compared with controls (odds ratio at 12 months 1.21; 95% CI 0.66–2.22) [20, 32, 46]. Two population-based studies accessed SEER-Medicare linked data to evaluate the association between ADT exposure and dementia. The first included 16,888 men with PC, with 2397 undergoing treatment with ADT [32]. In both a multivariable analysis and propensity score-matched analysis, there was an increased risk of Alzheimer’s disease associated with ADT exposure (hazard ratio [HR] 1.66; 95% CI 1.05–2.64 and HR 1.88; 95% CI 1.10–3.20, respectively). The second study included 9272 men with PC, with 1826 receiving ADT. The authors found that ADT was associated with an increased risk of dementia. However, the absolute risk occurred at 5 years (HR 2.17; 95% CI 1.58–2.99; 4.4% absolute risk at 5 years) [46]. A population-based study using the Korean National Health Insurance Service database analyzed the data on ADT and cognitive dysfunction between 2008 and 2015 in the Korean PC population, excluding patients with a previous diagnosis of cognitive dysfunction, dementia, or cerebral event history (*N* = 35,401). The authors reported a statistically significant association between ADT and the risk of cognitive dysfunction (HR 1.169; *P* = 0.002) [47].

Taken together, the emerging data suggest that the risk of ADT-associated cognitive disorders may vary in patients with PC, and calls for uniform methods of assessment coupled with a recognition of diverse genetic, societal, and comorbid features that may influence cognitive function. Further, methodological differences across studies likely contribute to conflicting results [20, 48, 49]. One important issue is the inconsistent definition of cognitive impairment [40] and the use of different measures of cognitive function [40]. Furthermore, small sample sizes, few prospective longitudinal studies, and short (<12 months) follow-up times can all potentially impact statistical conclusion validity. Different studies also employ varied ways of measuring and adjusting for symptoms, such as fatigue, pain, declining physical activity, reduced muscle mass, cardiovascular morbidity, and mood disturbance, that can commonly occur concurrently with PC and dementia [50], and may indirectly affect cognitive function. Age, disease status, and comorbidity may also affect cognitive function [51].

The studies we discuss here are either PCTs or population-based retrospective studies. Retrospective studies can facilitate the collection of large amounts of information over a short time period, but data availability can be a limitation (Table 1). Prospective clinical trials allow the establishment of specific clinical endpoints with reduced bias; however, patient data collected under specific trial conditions may not align with real-life disease management. Notably, the PCTs reviewed here included far more specific and tailored measurements of cognitive function than the retrospective studies (Table 1).

## Second-generation AR-targeted therapies: effects on CNS and cognitive function

### Enzalutamide

The second-generation AR inhibitor enzalutamide is currently approved for the treatment of mCRPC both before and after chemotherapy, and was approved for use in patients with nmCRPC in July 2018 [12, 28, 52]. It is generally well tolerated, but caution is advised in patients who have a history of seizure [13]. Data from animal models show that enzalutamide crosses the blood–brain barrier [53–55], in which it may cause the inhibition of the gamma-aminobutyric acid-gated chloride channel [54, 56], with resulting CNS effects, including lowering of the seizure threshold [54, 56, 57]. In animal models, convulsions were shown to be a dose-dependent toxic effect of enzalutamide at doses administered above the clinical therapeutic range [58]. An increased risk of seizure with enzalutamide has been observed in clinical trials, generally in association with higher than recommended (daily 160 mg) doses, or with comedications or conditions that could lower the seizure threshold [22, 23, 59–62]. In phase 3 studies of enzalutamide in the mCRPC population before and after chemotherapy, which carefully selected for patients at lower risk, seizures occurred in 0.6% in the post-chemotherapy setting, and 0.1% in the pre-chemotherapy setting [62, 63]. In addition to seizure, the cognition-impairing adverse event, fatigue, was attributed to enzalutamide (34–36% of patients); falls were also noted (11 events per 100 patient-years) as these may be associated with dizziness [62, 64]. Although seizure risk is low in properly dosed patients, its incidence in controlled trial settings highlights the potential for enzalutamide to penetrate the CNS (and plausibility of effects on cognitive function), serving as an uncommon example of this pharmacologic effect on the blood–brain barrier.

Additional studies describe CNS-related adverse effects associated with enzalutamide. The phase 2 TERRAIN trial randomized men with mCRPC to treatment with enzalutamide or bicalutamide [65]. Enzalutamide was associated with greater fatigue than bicalutamide (28% vs. 20%, respectively), which was offset by significantly better disease control [65]. A meta-analysis comparing rates of cognitive decline and mood disturbance in patients treated with enzalutamide and abiraterone acetate extracted data from the phase 3 pre- and post-chemotherapy studies, PREVAIL, AFFIRM, COU-302, and COU-301 [66]. The analysis found a statistically significant higher risk of anxiety, insomnia, headache, and restless leg syndrome in patients treated with enzalutamide vs. placebo, which was not reported in patients treated with abiraterone acetate vs. placebo [66].

The multicenter real-world REAAcT study (NCT02663193) assessed the respective tolerability of initiating enzalutamide and abiraterone acetate in patients with mCRPC [67]. More adverse events were reported with enzalutamide than with abiraterone acetate. Neuropsychiatric events specific to enzalutamide in this analysis included amnesia, “cognitive disorders” (not otherwise specified), memory impairment, and confused state. Four patients on enzalutamide and one on abiraterone acetate also showed clinically meaningful cognitive decline. Differences in fatigue were also noted more often with enzalutamide; the Functional Assessment of Chronic Illness Therapy Fatigue (FACIT-Fatigue) scale showed a median change of −4 with enzalutamide (compared with 0 for abiraterone acetate), and 26% of patients on enzalutamide showed fatigue-related adverse events, compared with 8% on abiraterone acetate (mean change −4, 95% CI −6.61 to −1.39) [67]. When studying clinically important differences (CID) in QoL measures, Cella et al. calculated a minimal CID for FACIT-F (fatigue) of 3 [68]. Initial results from the observational AQUARiUS study on fatigue and cognition in patients with mCRPC treated with enzalutamide and abiraterone acetate showed less favorable cognitive outcomes with enzalutamide, compared with abiraterone (measured by mean change from baseline) [69]. Significant differences favoring abiraterone acetate over enzalutamide were reported in the perceived cognitive impairments subscale of the FACT-Cog (4.67 [95% CI 1.20 to 8.13; *P* = 0.009]; 6.60 [95% CI 2.73 to 10.48; *P* = 0.001]; and 6.64 [95% CI 0.84 to 12.43; *P* = 0.025] at months 1, 2, and 3, respectively) and in the subscales for “impact on QoL” at month 1 (1.36 [95% CI 0.00 to 2.71; *P* = 0.050]), and “comments from others” at month 3 (1.53 [95% CI 0.44 to 2.62; *P* = 0.007]). Significant differences favoring abiraterone acetate were also observed using the European Organisation for Research and Treatment of Cancer Quality of Life Questionnaire-Core 30 (EORTC QLQ-C30) for cognitive functioning across all time-points (6.10 [95% CI 0.92 to 11.28; *P* = 0.021]; 9.75 [95% CI 3.06 to 16.44; *P* = 0.005]; and 11.82 [95% CI 0.84 to 22.79; *P* = 0.035] at months, 1, 2, and 3, respectively). Greater levels of fatigue were also reported with enzalutamide using the brief fatigue inventory short form assessment tool (usual level of fatigue 1.17 [95% CI −2.13 to −0.22; *P* = 0.017]; and −1.41 [95% CI −2.74 to −0.08; *P* = 0.038] at months 2 and 3, respectively; fatigue interference −0.99 [95% CI −1.83 to −0.15; *P* = 0.021]; and −1.20 [95% CI −2.31 to −0.08; *P* = 0.036] at months 2 and 3, respectively; at month 3 for “your fatigue right now” −1.41 [95% CI −2.55 to −0.26; *P* = 0.017]; and “your worst level of fatigue” −1.63 [95% CI −2.98 to −0.28; *P* = 0.019]). Its real-world setting is a strength of the AQUARiUS study, but limitations include the evaluation of cognitive function by PRO data (which was not always collected consistently), with no baseline. More mature data from a larger population is needed [69]. The UPWARD single-arm, open-label study investigated seizure risk in patients with mCRPC and seizure risk factors who received enzalutamide in institutional practice (*n* = 366 [of 423 patients in total]) over a 4-month study period [70]. The authors calculated an incidence of confirmed seizure of 2.6 per 100 patient-years. In comparing this to the seizure rate of 2.8 per 100 patient-years noted in a large retrospective analysis of US patients (selected from MarketScan Commercial and Medicare Supplemental Databases) with mCRPC and similar seizure risk factors but no exposure to enzalutamide [71], the authors concluded that that enzalutamide did not increase seizure risk in this patient population [70].

Enzalutamide has also been investigated in patients with nmCRPC and prostate-specific antigen doubling time (PSADT) ≤10 months in the phase 3 PROSPER trial [28]. Hussain and colleagues reported that adverse events were consistent with the established safety profile of enzalutamide. The most common adverse event in patients receiving enzalutamide was fatigue (33% [303 of 930 patients] vs. 14% [64 of 465] patients). Mental impairment disorders (5% [48/930] vs. 2% [9/465] patients) were reported as occurring more frequently (by ≥2 percentage points) with enzalutamide than placebo (numerical difference only, *P* values are not reported). Convulsion was reported in three patients in the enzalutamide group (<1%) vs. 0 patients in the placebo group and was considered serious and drug-related in all three cases. Falls were also reported as an adverse event in PROSPER (11.0% [106/930] vs. 4.0% [19/465], respectively); however, falls were not associated with dizziness or seizure in the AFFIRM, PREVAIL, or PROSPER studies [12, 28]. The PREVAIL study reported a higher incidence of falls in elderly patients randomized to enzalutamide compared with placebo (19.2% [61 of 317] vs. 7.9% [23 of 292] patients), but the authors suggest that this might be fatigue related [72].

### Abiraterone acetate

The androgen synthesis inhibitor abiraterone acetate is approved for the treatment of mCRPC [9]. It targets CYP17A to inhibit residual androgen synthesis in the tumor and adrenal gland [53]. This also results in mineralocorticoid level aberrations that contribute to a number of adverse events, including hypokalemia, hypertension, and fluid retention [25]. To attenuate the incidence and severity of mineralocorticoid excess, abiraterone acetate is co-administered with low-dose prednisone [25]. At the low dosage used, prednisone-induced adverse effects, such as mood disorders and cognitive changes, as well as bone loss and immunosuppression, should be uncommon [9, 25]. In pivotal trials for abiraterone acetate (COU-AA-301 and -302), the frequency of fatigue was similar in patients treated with abiraterone acetate plus prednisone, vs. prednisone alone (44% [346/791] vs. 43% [169/394] and 39% [212/542] vs. 34% [185/540], respectively, in the two trials; *P* values for comparisons not calculated); patients with a history of seizures were not excluded from these trials, and seizure events were not reported [73, 74].

The available evidence suggests that CNS effects are less prevalent with abiraterone than with enzalutamide. In a preliminary analysis of an ongoing phase 2 trial of abiraterone and enzalutamide in patients with mCRPC (NCT02125357), fewer patients in the abiraterone acetate vs. enzalutamide group showed worsening of depression scores (as measured by the Patient Health Questionnaire [PHQ-9]; 4% vs. 19%, respectively; *P* = 0.03), with a trend for fewer patients with worsening cognitive function in this group as measured by the Montreal Cognitive Association (MoCA) test (5% vs. 19%; *P* = 0.20) [27]. These observations from prospective clinical trials are supported by real-world data showing that mCRPC patients treated with abiraterone acetate were less likely to experience a CNS event (defined by the authors as a post-index healthcare claim from the Truven Health MarketScan Research databases containing one or more diagnosis codes for amnesia or memory impairment, anxiety, ataxia, cognitive disorders, confusion, convulsions, disturbance in attention, dizziness, falls, fatigue/asthenia, hallucinations, headaches, insomnia, pain, paresthesia, seizures, weakness, or other CNS disorders [23]) than patients who received enzalutamide (39.5% vs. 46.0%, respectively at 12 months; *P* = 0.0036); on multivariate analysis adjusted for the presence of metastases, patients treated with abiraterone acetate had a 20% reduction in their 12-month risk of CNS events compared with those receiving enzalutamide [23]. In a real-world study of patients with mCRPC conducted in Japan, fatigue was reported by 19.4% of patients treated with abiraterone acetate and 32.3% of those treated with enzalutamide [75]. As previously noted, the REAAcT study found that fatigue and neuropsychiatric adverse events were reported less often with abiraterone than with enzalutamide; CNS effects specific to abiraterone included cerebrovascular accident, presyncope, and spinal cord compression [67]. A meta-analysis found that neuropsychiatric adverse events were more prevalent with enzalutamide compared with abiraterone acetate plus prednisone; patients treated with enzalutamide had a statistically significant higher risk of restless legs syndrome, anxiety, headache, and insomnia compared with control. Both enzalutamide and abiraterone acetate plus prednisone also showed a significant increase in risk of falls, compared with control (*P* < 0.05) [76]. The real-world Canadian Observational Study in Metastatic Cancer of the Prostate study found no cognitive decline over time in patients with mCRPC on abiraterone acetate plus prednisone (mean baseline Montreal Cognitive Assessment score was 25.2; subsequent assessments scored above 26, with a mean absolute change from baseline of <1) [77].

### Apalutamide

The second-generation AR inhibitor apalutamide was approved in February 2018 for treatment of nmCRPC [3, 6, 9, 52]. Apalutamide blocks AR nuclear translocation or binding to AR elements by selectively binding to the ligand-binding domain of the AR [72, 78]; it can be associated with effects that suggest CNS penetration, including falls. In a phase 2 open-label trial, fatigue required apalutamide dose reduction in 4% of patients and treatment discontinuation in another 4% [78].

The phase 3 SPARTAN (NCT01946204) trial investigated the addition of apalutamide to ADT in patients with nmCRPC and a PSADT of ≤10 months [24]. A higher number of mental impairment disorders was seen in the apalutamide group compared with placebo (5.1% [41 of 803 patients] vs. 3% [12 of 398], respectively; numerical difference only, *P* values were not reported); higher numbers of patients in the apalutamide group also reported fatigue (30.4% [244/803] vs. 21.1% [84/398], respectively) and dizziness (9.3% [75/803] vs. 6.3% [25/398], respectively). Falls were also reported as an adverse event (15.6% [125/803] vs. 9.0% [36/398], respectively; numerical difference only, *P* values were not reported); however, in SPARTAN, falls were not associated with dizziness or seizure [15]. Multivariate analysis of falls/fractures in apalutamide-treated patients identified older age, poor Eastern Cooperative Oncology Group performance status, history of neuropathy, and α-blocker use with a higher rate of falls [79].

Although the selection criteria excluded patients with a history of seizure or predisposing conditions, two seizures were reported in patients in the apalutamide group; these were considered by the investigators to be related to the trial regimen [24, 80]. Of note, neither the SPARTAN nor PROSPER studies conducted standardized cognitive testing, leaving the assessment of cognitive effects confined to the Common Terminology Criteria For Adverse Events [24, 28, 80], which were not established for the measurement of cognitive function, but leave such determinations to investigator assessment of broader categories of effects.

The phase 3 ATLAS (NCT02531516) trial of apalutamide in patients with nmCRPC and in men with local high-risk or locally advanced PC receiving primary radiotherapy is ongoing; however, patients with a history of seizure or a predisposing condition are excluded, and effects on cognition are not being specifically evaluated [81].

### Darolutamide

**Fig. 1:**
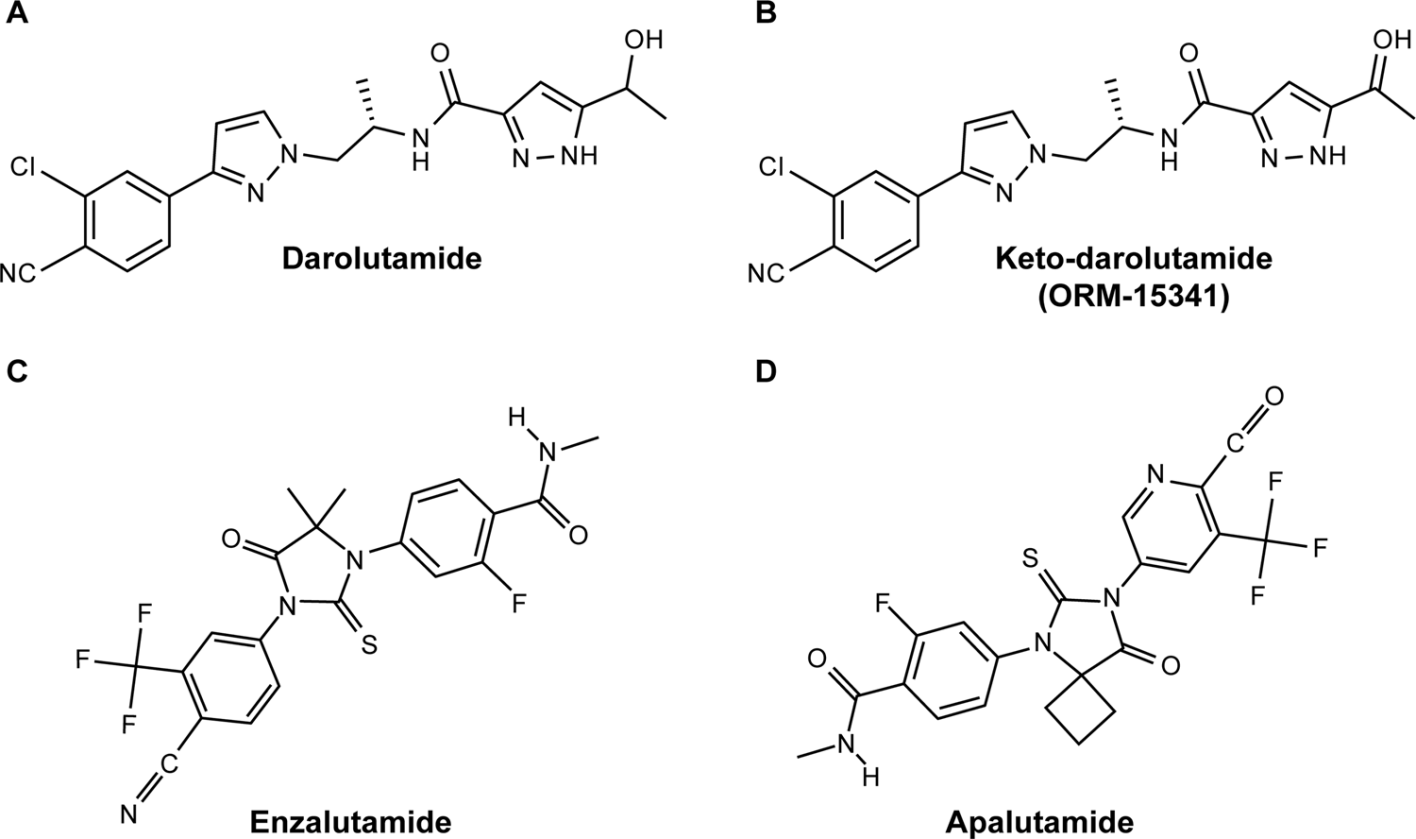
Structures of second generation antiandrogens. Darolutamide (**a**) and its metabolite keto-darolutamide (**b**) are structurally different to the second-generation AR inhibitors enzalutamide (**c**) and apalutamide (**d**). [**a** and **b** reproduced from Moilanen et al. 2015: https://www.nature.com/articles/srep12007 (drug names updated) [54]; **c** and **d** reproduced from PubChem [93, 94]].

Darolutamide is a structurally distinct AR antagonist (Fig. 1) [54]. Darolutamide and its main circulating metabolite, keto-darolutamide, block the growth of PC cells by inhibiting AR function and testosterone-induced nuclear translocation; the inhibition constant values (the concentration required to produce half-maximum inhibition) for darolutamide and keto-darolutamide were 11 nM and 8 nM, respectively, lower than those for enzalutamide (86 nM) and apalutamide (93 nM) [54, 82].

**Fig. 2:**
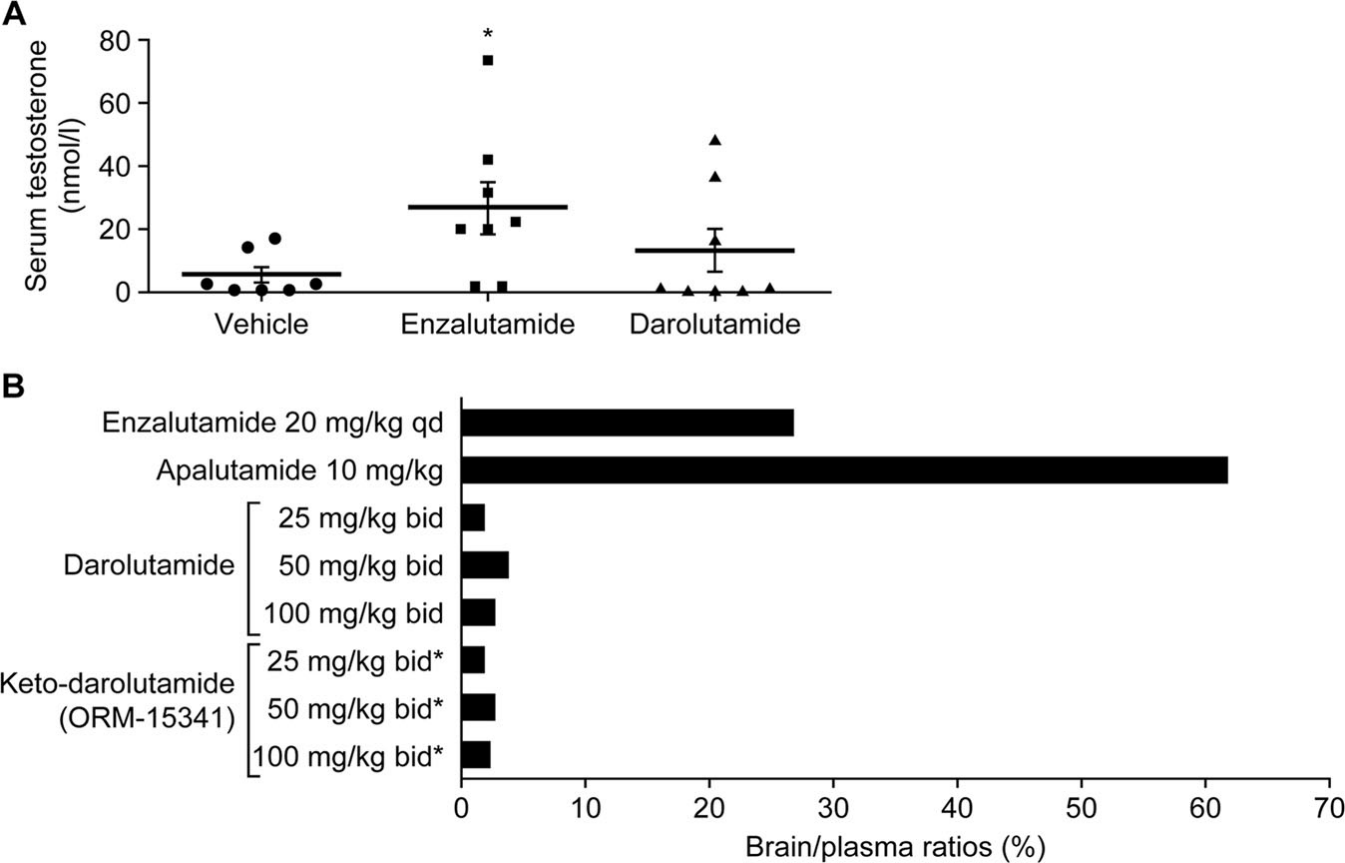
Comparison of second generation antiandrogens in preclinical studies. **a** Serum testosterone levels (nmol/L ± SEM) of mice models of VCaP tumors after oral treatment with vehicle, enzalutamide (20 mg/kg, qd), or darolutamide (50 mg/kg, bid) for 3 weeks (*n* *=* 8). *P *<* 0.05 vs. vehicle. **b** Mean brain–plasma ratios (%) in mice after oral treatment with darolutamide (25, 50, or 100 mg/kg, bid for 7 days), enzalutamide (20 mg/kg, qd for 7 days; *n* *=* 5), or apalutamide (a single dose of 10 mg/kg; *n* *=* 3). *Evaluated from different concentrations of darolutamide. *bid* twice daily, *qd* once daily, *SEM* standard error of the mean. [Reproduced from Moilanen, et al. 2015 [54] https://www.nature.com/articles/srep12007 (drug names updated)].

In preclinical studies, darolutamide demonstrated much lower blood–brain barrier penetration relative to enzalutamide and apalutamide, with brain–plasma drug ratios of 1.9–3.9% (1.9–2.8% for keto-darolutamide), compared with 27% for enzalutamide, and 62% for apalutamide (Fig. 2) [54]. A separate preclinical study demonstrated that blood–brain barrier penetration of darolutamide was ~10% that of enzalutamide [55]. In addition, because of its relatively low blood–brain barrier penetration, preclinical data suggest that darolutamide does not increase serum testosterone levels, unlike the AR inhibitors, which can increase serum testosterone levels through stimulation of luteinizing hormone signaling (Fig. 2) [54].

Both the phase 1 open-label ARAFOR and the phase 1/2 open-label dose escalation/expansion ARADES clinical studies of darolutamide included patients with a history of seizures [82–84]. In ARAFOR, conducted in men with chemotherapy-naïve mCRPC, 91% of adverse events were mild or moderate in severity (grade 1 or 2), and fatigue was the most common adverse event (13% of patients) [83]. The ARADES study was conducted in men with mCRPC (before or after chemotherapy); the most common treatment-related adverse events were fatigue or asthenia (12% of patients), with one patient experiencing grade 3 fatigue and asthenia that was considered related to darolutamide therapy [82]. No seizures were reported in either study [85].

The phase 3 ARAMIS trial of darolutamide plus continuing ADT in men with nmCRPC and PSADT ≤10 months included patients with a history of seizures or any condition predisposing to seizures. Darolutamide was not associated with increased rates of seizures, falls, or cognitive disorder compared with the placebo arm (both plus ADT), and demonstrated comparable incidences of AEs with placebo (falls, 4.2% [40/954] vs. 4.7% [26/554]; seizures, 0.2% [2/954] vs. 0.2% [1/554]; dizziness, 4.5% [43/954] vs. 4.0% [22/554]; cognitive disorder, 0.4% [4/954] vs. 0.2% [1/554]; memory impairment, 0.5% [5/954] vs. 1.3% [7/554]—all respectively, darolutamide vs. placebo; *P* values for comparison were not calculated), with the exception of fatigue and asthenic conditions, 15.8% with darolutamide (151/954) vs. 11.4% with placebo (63/554) [86]. These results suggest a favorable safety profile for darolutamide in relation to CNS-related adverse events.

The efficacy and safety of darolutamide is being further investigated in the ongoing phase 3 ARASENS clinical trial (NCT02799602) in patients with metastatic hormone-sensitive PC [87].

### Clinical implications

CNS complications associated with systemic anticancer therapies have the potential to influence the ability of patients to make informed decisions about treatment and participate in occupational or leisure activities, and can reduce QoL [88, 89]. Given this, it is imperative for practitioners to be aware of the symptoms and incidence to effectively monitor patients for these complications and refer them to appropriate specialists for a thorough evaluation when needed. The National Comprehensive Cancer Network (NCCN) guidelines for Survivorship (Cognitive Function) suggest some simple screening tools that can be performed by the primary treating clinician, followed by referral for neuropsychological examination [90]. Other potential first-line interventions include cognitive rehabilitation, exercise, psychotherapy, and symptom validation [91, 92].

## Conclusions

AR-directed therapies for PC are associated with CNS effects in some patients. Changes in cognitive function are most readily identified when a pretreatment baseline has been established, and when robust clinical neuropsychological tests are used to evaluate patients, necessitating a multidisciplinary approach to case management. Research in this area has used a variety of tests that complicate our understanding of the nature, incidence, and risks for these treatment-related, adverse effects. Nevertheless, the impact of AR-directed therapies on cognitive function and the CNS need to be identified and managed to ensure that patient QoL is maintained. At present, available data indicate that agents such as darolutamide and abiraterone acetate may be associated with a lower risk of CNS-related adverse events than enzalutamide and apalutamide. However, as no head-to-head randomized studies of these agents have been conducted, continued investigation is necessary to fully characterize their CNS effects. As recognition of the CNS effects of PC cancer treatment has grown, the number of ongoing studies evaluating these effects has increased. Data from recently completed and ongoing studies (Table 2) will facilitate better understanding of the effects of AR-directed agents and PC itself on the CNS. Clarifying the relationship between AR-targeted agents, direct AR antagonist/inhibitor activity in the CNS, and CNS effects will enable clinicians and patients to make informed decisions regarding therapies and support the development of management strategies for patients with PC treatment-related CNS effects and cognitive dysfunction.

**Table 2:** Summary of ongoing clinical trials evaluating CNS effects of androgen ablation therapy in men with prostate cancer.

NCT number/study name	Study design	Primary objectives	Secondary objectives	Estimated enrollment	Primary completion date	Potential limitations
NCT03016741	Prospective, phase 4, interventional, randomized, parallel assignment study to investigate cognitive function in men with mCRPC treated with abiraterone acetate – or enzalutamide (+ADT)	Cognitive function defined by overall Cogstate score and Cogstate module scores for each domain (measured at baseline, 3, 6, and 12 months)	EORTC QLQ-C30, FACIT- Fatigue, FACT-Cog, depression by PHQ-9, instrumental activities of daily living by Texas Functional Living Scale (measured at baseline, 3, 6, and 12 months), SNPs associated with cognitive function, imaging assessed by MRI	100	August 2020	A well-designed trial that may answer some important questions on cognitive function
NCT03927391/ REDOSE	Prospective, phase 4, randomized, parallel assessment study to investigate reduced enzalutamide dose on cognitive side effects in frail patients with mCRPC	Change in fatigue after 6 weeks of treatment, as measured by FACIT-Fatigue	Decrease in fatigue after 12 and 24 weeks of treatment; cognitive impairment and its impact on QoL (measured by FACT-Cog and MoCA); changes in depression score (measured by GDS-15; all at 6, 12, and 24 weeks)	50	May 2021	A well-designed trial, although sample size is not large. QoL is measured by PRO only; MoCA is not a comprehensive test battery
NCT03016741/ COG-CaP	Prospective, randomized trial comparing cognitive effects of enzalutamide and abiraterone acetate in men with mCRPC	Compare cognitive function and associated mediators in men with mCRPC or mHSPC during treatment with enzalutamide (mCRPC only) or abiraterone acetate (mCRPC or mHSPC)	Identify characteristics associated with change in cognitive function in men with mCRPC, during AR-directed therapy. Compare QoL and associated factors of men with mCRPC during enzalutamide/abiraterone acetate therapy	100	August 2020	Trial is not randomized, and does not appear to include formal neurocognitive testing
NCT02907372/COG-PRO	Prospective, open-label, interventional, diagnostic study on the impact of abiraterone acetate and enzalutamide in elderly patients with mCRPC	The proportion of elderly patients who will experience increased cognitive concerns (at least for one cognitive complaint) by questionnaires (3 months after initiation of treatment)	Questionnaires on cognitive function, QoL, anxiety/depression, fatigue, observance of treatment, and autonomy after 12 months	222	December 2019	This study uses questionnaires (rather than formal neurocognitive testing), obtained at 3 months, not baseline
NCT00579072	Observational case–control study to assess the impact of androgen ablation therapy (leuprolide acetate, bicalutamide, goserelin, or degarelix acetate) on cognitive functioning and functional status in elderly men (aged ≥65 years) with prostate cancer	Describe changes in cognitive functioning over 6 months in men who are about to start androgen ablation therapy vs. men with neuroendocrine differentiation and no medical indication to start comparison androgen ablation therapy (3 years)	Differences in cognitive functioning of patients with comparison androgen ablation therapy vs. those who are hormone naïve or who received androgen ablation therapy, regionally specific differences in brain activity mediated by testosterone, and differences in neuropsychological performance	280	December 2019	This study uses questionnaires (rather than formal neurocognitive testing)
NCT03124615/EFFECT	Phase 2, open-label, interventional study in patients with mCRPC who have started enzalutamide treatment and have grade 3 fatigue and/or cognition complaints to determine whether dose reduction of enzalutamide will lead to an improvement in symptoms while maintaining active drug levels	The proportion of patients who have an improvement in cognition/fatigue symptoms after 12 months	–	47	December 2020	Cognitive function alterations are patient-reported only

*ADT* androgen deprivation therapy, *AR* androgen receptor, *EORTC QLQ-C30* European Organization for Research and Treatment of Cancer Quality of Life Questionnaire-Core 30, *FACIT-Fatigue* Functional Assessment of Chronic Illness Therapy Fatigue Subscale, *FACT-Cog* Functional Assessment of Cancer Therapy-Cognitive, *GDS* geriatric depression scale, *HRQoL* health-related quality of life, *mCRPC* metastatic castration-resistant prostate cancer, *mHSPC* metastatic hormone-sensitive prostate cancer, *MoCA* Montreal Cognitive Association Montreal Cognitive Association, *MRI* magnetic resonance imaging, *PHQ-9* Patient Health Questionnaire, *PRO* patient-reported outcome, *QoL* quality of life, *SNP* single nucleotide polymorphism
